# In Silico Identification and Conservation Analysis of B-cell and T-Cell Epitopes of Hepatitis C Virus 3a Genotype Enveloped Glycoprotein 2 From Pakistan: A Step Towards Heterologous Vaccine Design

**DOI:** 10.5812/hepatmon.9832

**Published:** 2014-06-01

**Authors:** Aqsa Ikram, Sadia Anjum, Muhammad Tahir

**Affiliations:** 1 Atta-Ur-Rahman School of Applied Biosciences, National University of Sciences and Technology, Islamabad, Pakistan

**Keywords:** Hepatitis C Virus, Structural Homology, Protein, Computer Simulation, Insilco, Immunogenicity

## Abstract

**Background:**

Hepatitis C virus (HCV) is known for the eminent global disease burden responsible for encumbering public health. Development of an effective vaccine is the major need of the day; however, several obstacles loom ahead of this objective. One of the major barriers is that as a RNA virus, it mutates rapidly resulting in high sequence divergence and several viral isolates in the world. Theglycoprotein 2 (gpE2) is the primary component of HCV envelope with direct interaction with the host cell surface receptors; it is an indispensable target of neutralizing antibodies and hence, should be a fundamental component of vaccine design.

**Objectives:**

This study focused on B-cells and T-cells epitopes prediction in HCV gpE2, particularly in 3a genotype, in Pakistan and identification of the conserved epitopes among various 3a isolates at global level, principally conserved across HCV major genotypes.

**Materials and Methods:**

Epitope finding was done by using online available bioinformatics tools including Immune Epitope Database (IEDB), ProPred-I, and ProPred. Conservation of these epitopes was found by aligning selected gpE2 sequences using MultAlin online software and conservancy analysis tool available at IEDB.

**Results:**

Many B-cell and T-cell epitopes predicted in gpE2 were found conserved among HCV 3a genotypes whereas few were conserved in other genotypes anticipating these epitopes as potential candidates of producing strong B-cell and T-cell response against HCV 3a and other genotypes.

**Conclusions:**

HCV gpE2 is an ideal target for HCV vaccine. Prediction of epitope immunogenicity and characterization on the basis of peptide sequences will be significantly helpful for development of a heterologous vaccine against HCV variants.

## 1. Background

Hepatitis C virus (HCV) is a serious health problem with over 200 million people worldwide at risk of infection (1-3). In most patients, HCV remains asymptomatic for years after infection and the patients are often unaware of their infection until it becomes too late for effective treatment. About 20% to 50% of infected individuals develop progressive liver disease that ultimately leads to liver cirrhosis, liver failure and hepatocellular carcinoma (4-6). According to World Health Organization (WHO) report, HCV accounts for over 350000 deaths per year worldwide with anticipated three to four million new infections each year. Countries with the highest rates of chronic infection are Egypt (22%), Pakistan (4.8%) and China (3.2%). Unsafe medical practices in Pakistan are the main cause of enormous spread of HCV (7). More than ten million people are living with this fatal disease in Pakistan (8, 9), which is an alarming number.

The HCV genomic RNA consists of a long open reading frame of over 9024 nucleotides and relatively short untranslated regions (UTR) at the 5′ and 3′ ends. The 5′ and 3′ UTR contain cis-acting RNA elements necessary for HCV polyprotein translation and RNA replication. Thepolyprotein is eventually cleaved by cellular peptidases and viral proteases to produce structural (core as well as envelope glycoproteins 1 [gpE1] and 2 [gpE2]) and nonstructural components (NS2, NS3, NS4, and NS5A) (10).

Unfortunately, there is no available vaccine against HCV. Efforts done for developing HCV vaccine have been hindered by several factors including the prone to high-error replication of HCV (11), *gpE2* gene as the most variable component of the viral genome, lack of suitable animal models and the absence of well-established in vitro knowledge of protective immunity (12). Recent studies had shown that CD4+ and CD8+ T-cell responses are essential in the control of acute HCV infection and it is suggested that neutralizing anti-HCV antibody responses play a significant role in the natural clearance of HCV infection. Novel vaccines based on molecular technology for eliciting proper immune response against HCV, including both broadly neutralizing antibodies and effective T-cell response, are the part of current discussions in literature (13).

Evidence from clinical and experimental studies on human and chimpanzees suggests that HCV gpE2 is a key antigen for developing a vaccine against HCV infection (14). Broadly neutralizing antibodies are usually directed against conformational epitopes within gpE2 (15). HCV induces a strong antibody response to its gpE2. Since gpE2 binds to B cells via CD81, antibodies to gpE2 are anticipated to block binding of HCV to cells providing a protective shield against HCV infection (16, 17). In addition to antibodies, HCV gpE2-specific T cells are critical for infected cells clearance from HCV (12). Designing of conserved epitopes in highly diverse gpE2 that are capable of eliciting protective antibodies and T-cell response as well as generating antigen-specific memory cells is a momentous challenge of the present decade (18). Immunogenic epitopes used as peptide vaccines or polytope DNA vaccine are promising approach for HCV with high mutation rates. However, appropriate design and primary in silico analysis is an essential prerequisite before commencing costly transgenic animal studies (19, 20). Computationally predicted CD8+ epitopes had shown encouraging delayed-type hypersensitivity response in vaccinated mice (21). Accurate prediction of peptide immunogenicity and characterization of relation between peptide sequences and peptide immunogenicity will be greatly helpful for vaccine designs and understanding of the immune system (22).

## 2. Objectives

The present study aimed to locate conserved B-cell and T-cell epitopes in HCV 3a genotype *gpE2* gene cloned from HCV infected patients from Pakistan by using online bioinformatics tools including Immune Epitope Database (IEDB), ProPred-I, and ProPred. The conservation of predicted epitopes by these tools was compared among Pakistan, Asia, and the world population affected by HCV 3a and other genotypes. In addition, a specific criterion for epitope conservation was proposed in this study that might help to find out specific epitopes not only in HCV *gpE2* but also in other important immunogenic genes.

## 3. Materials and Methods

### 3.1. Genotype 3a Envelope Glycoprotein 2 Gene Consensuses Sequence

The HCV 3a genotype consensuses sequence was done by aligning 24 different *gpE2* sequences retrieved from gene bank in Pakistan (Table 1). ClustalW and MultAlin online available software were used for sequence alignment. The consensus sequence “*E2PK*”was then used for further applications.

**Table 1.** Sequences of HCV Envelop Glycoprotein 2 gene From Various Countries Included in This Study ^a,b^

Country	G-1	G-2	G-3	G-4	G-5	G-6	Total	-
**Germany**	4	-	1	-	-	-	5	AJ132996 (1a), AJ132997 (1a), EU155382 (1b), EU155381 (1b), X76918
**UK**	10	-	18	-	2	-	30	
**Switzerland**	5	-	1	-	-	-	6	EU255960 (1b), EU155356 (1b) EU255957 (1a), EU255958 (1a), EU255927 (1a)
**Ireland**	8	-	-	-	-	-	8	
**Denmark**	-	-	1	-	-	-	1	GU814263 (3a)
**Spain**	2	2	-	2	-	-	6	AJ851228, AM910652 (1g), FN666428 (2q), FN666429 (2q)
**Indonesia**	1	-	1	-	-	1	3	D14853 (1c), D63821 (3K), D63822 (6g)
**Japan**	4	4	2	-	-	-	10	
**Turkey**	2	-	-	-	-	-	2	AF483269 (1b), Af483269 (1b)
**Thailand**	-	-	-	-	-	3	3	D84262 (6b), DQ835760 (6f), DQ835761 (6j)
**Egypt**	-	-	-	4	-	-	4	DQ988073 (4a), DQ988074 (4a), DQ988075 (4a), DQ988076 (4a)
**Brazil**	5	-	-	-	-	-	5	EF032886 (1a), EF032887 (1a), EF032888 (1a), EF032892 (1b), EF032893 (1b)
**Pakistan**	1	-	23	-	-	-	24	
**China**	1	3	3	-	-	2	9	
**India**	1	-	10	-	-	-	11	
**USA**	12	5	3	2	-	-	22	
**Australia**	-	-	1	-	-	-	1	AF046868 (3a)
**South Africa**	-	-	-	-	1	-	1	AF064490
**Vietnam **	-	-	-	-	-	3	3	D84263 (6b), D84264 (6b), D84265 (6b)
**Canada **	-	-	-	-	-	2	2	EF424625 (6q), EF424623 (6p)
**Portugal**	-	-	-	3	-	-	3	FJ025854 (4b), FJ025855 (4b), FJ025856 (4b)
**Italy**	-	-	15	-	-	-	15	
**Total**							174	

^a^ Abbreviation: G, genotype.

^b^ Accession numbers of sequence less than seven from a country are given in the table. For countries more than six sequence, accession numbers are given here: United Kingdom, GQ370157(1a), GQ370154(1a), GQ370155(1a), GQ370157(1a), GQ370159(1a), GQ370164 (1b), GQ370163 (1b), GQ370162 (1b), GQ370165 (1b), GQ370170 1b), GQ356441(3a), GQ356442(3a), GQ356431(3a),GQ356437(3a), GQ356427(3a), GQ356424(3a), GQ356477(3a), GQ356452(3a), GQ356499(3a), GQ356486(3a), GQ356456(3a), GQ356471(3a), GQ356571(3a), GQ356559 (3a), GQ356565(3a), GQ356558(3a), GQ356554(3a), and GQ356549(3a); United States, AF009606, AF011751, AF011752, AF011753, DQ838744, DQ838745, DQ889259, DQ889257, AY695437, AY956463, AY956466, AY956469, JF779679(1/2b), NC_00923(2a), DQ364460(2b), DQ430815(2b), DQ430817(2b), AY956467(3a), DQ430819(3a), and DQ430820(3a);Italy, GU814263, GQ356425, GQ356435, GQ356438, GQ356440, GQ356442, GQ356444, GQ356462, GQ356465, GQ356769, GQ356770, GQ356771, GQ356777, GQ356778,andGQ356779;Ireland, AB154198(1b), AB154201(1b), AB154204(1b), AB154206(1b), AB154191(1b), AB154200(1b), Ab154199(1b), AB154193(1b), AB154191(1b), AB154200(1b), Ab154199(1b), and AB154193(1b); Japan, AB049097(1b), AB049101(1b), D89872(1b), D90208(1b), AB047639(2a), AB047642(2a), AB661373(2b), AB661374(2b), D49374(3b), and D17763(3a);China, EU857431(1b), HQ639939(2a), HQ639943(2a), HQ639944(2a), HQ639941(3a), HQ639942(3a), HQ912953(3a), AY878650(6k), and AY878651(6k); India, HQ738645(3a), JN714194(3a), JQ717254(3a), JQ717255(3a), JQ717260(3a), JQ717256(3a), JQ717257(3a), JQ717258(3a), JQ717259(3a), and GQ275355(3a); Pakistan, GQ898898(1a), GU294484(3a), HM590012(3a), HM584120(3a), MT003(3a), GQ355940(3a), GQ355941(3a), GQ355942(3a), HQ108092(3a), HQ108093(3a), HQ108094(3a), HQ108095(3a), HQ108096(3a), HQ108097(3a), HQ108098(3a), HQ108099(3a), HQ108100(3a), HQ108101(3a), HQ108102(3a), HQ108103(3a), HQ108104(3a), HQ108105(3a), HQ08106(3a), and HQ108107(3a).

### 3.2. Programs and Databases

#### 3.2.1. The Protein Information Resource Database

PIR database The Protein Information Resource (PIR; http://pir.georgetown.edu/) database was employed to determine molecular weight, percentage of highly repeated amino acid, and the least repeated amino acid in the viral gpE2. This database is a computer-based method for the comparison of protein sequences, detection of distantly related sequences, and duplications within sequences.

#### 3.2.2. VaxiJen

VaxiJen, a bioinformatics tool, was used for analyzing antigenic property of HCV 3a genotype *gpE2* gene and its comparison with other HCV genes (Table 2). VaxiJen predicts each of the HCV proteins for antigenicity property. VaxiJen is the server for alignment independent prediction of protective antigen. It allows antigen classification based onthephysiochemical properties of proteins and uses autocross-covariance (ACC) transformation of protein sequences into uniform equal-length vectors. Antigenicity scores are shown in Tables 3, 4, 5 and 6.

#### 3.2.3. ProPred-I and ProPred

Promiscuous T-cell epitopes of HCV 3a genotype gpE2 were predicted for both class I and II MHC binding by using online immune informatics tools such as ProPred-I, and ProPred. ProPred-I and ProPred epitope prediction tools cover maximum number of Human Leukocyte antigens e.g. HLA. The ProPred-I is an online tool to identify and predict the Class IMHC binding regions in protein antigens (23). It predicts binding peptides for 47 alleles. This is a matrix-based method, which also allows the prediction of the standard proteasome and immune proteasome cleavage sites in an antigenic sequence. This server helps in identifying the promiscuous T-cell epitopes. ProPred server predicts Class II MHC-binding regions in an antigen sequence, using quantitative matrices proposed by Sturniolo et al. in 1999 (24).ProPred server allows predicting 57 allele-specific class II MHC-binding peptides. The server helps to determine promiscuous binding regions that are useful in selecting vaccine candidate.

#### 3.2.4. Hepatitis C Virus Database

Total of 173 nucleotide sequences were selected from Hepatitis C Virus Database (http:// www.hcvdb.org/) and Gen Bank (http://www.ncbi.nlm.nih/). These sequences were reported from Germany, Switzerland, Ireland, Denmark, Spain, Indonesia, Japan, Turkey, Thailand, Egypt, Brazil, Pakistan, China, India, United States, South Africa Australia, Vietnam, Canada, Portugal and Italy (Table 1).

#### 3.2.5. Epitope Conservation Analysis

The predicted B-call and T-cell epitopes of HCV 3a genotype *gpE2* from Pakistani isolates (E2PK) were subjected for conservation analysis from Pakistan, Asia, and all over the world. Conservation of these predicted epitopes was also rated for major HCV 1 to 6 genotypes worldwide. In case of T cell, only those epitopes that bind to maximum number of alleles were selected. The predicted epitopes of HCV 3a (E2PK) along with selected sequences of genotypes 3a (23 from Pakistan, 30 from Asia, and 50 from other countries) and genotypes 1 to 6 (70 from other countries) were submitted to epitope conservation analysis tool (IEDB). The epitopes with 80% to 100% conservancy were selected. Finally, all the selected conserved epitopes were analyzed for similarity with human proteome using Blast program (http://www.ncbi.nlm.nih.gov/BLAST/) to verify that these peptides will not trigger auto immunity.

## 4. Results

The HCV gpE2consensus sequence was developed using sequences reported from Pakistan. The consensus sequence was then used to predict various antigenic epitopes within this protein. Probable antigenic protein value for *gpE2* was identified by VaxiJen at 0.4 threshold level and was also compared with other HCV genes. The HCV gpE2 showed highest antigenicity value of 0.49 as compare to other HCV proteins (Table 2) introducing gpE2 as a potential candidate for HCV vaccine development.

**Figure 1.**
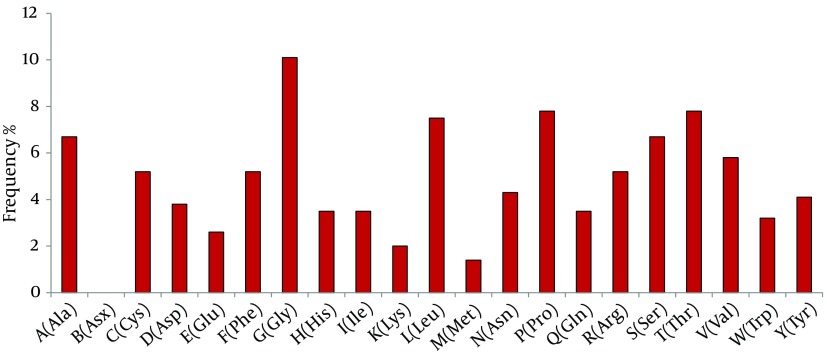
Percentage of Highly as Well asLeast Repeated Amino Acid Residues in Hepatitis CVirus Consensus Sequence of Envelope Glycoprotein 2 (E2PK)

The molecular weight of gpE2 was found to be 38042.92 KDa. Among the amino acids, glycine has the highest repetition rate followed by proline, leucine, threonine, serine, alanine, and valine (Figure 1). Repetitions of amino acid residues determine the probability of a particular proteinantigenicity. Proteins frequently containing cystine, leucine, and valine are expected to have more antigenic determinants. Seventeen B-cell epitopes were predicted by IEDB in gpE2 (Table 3).

Ninety-five epitopes in gpE2 (E2PK) were predicted against 57 class II MHC-specific alleles by ProPred-I. Among them, 30 epitopes were worth of discussion (Tables 4 and 5). Epitopes R1 to R3 were found to be ≥ 90% conserved and epitopes R4 to R6 showed ≥ 80% conservation among 3a and other genotypes. Other epitopes designated as Q1 to Q16 showed ≥ 90% conservation and epitopes P1 to P6 conservation was 80% among 3a population with no conservation in other genotypes (Tables 4 and 5).

Few epitopes were predicted against 47 class I MHC-specific alleles by ProPred. Among these epitopes M1 and M2 were conserved in HCV 3a and other genotypes (Table 6) whereas epitope M3 showed above 90% conservation only in 3a population.

To avoid the autoimmune response, all the predicted B-cell-binding and class I as well as II MHC-binding antigenic regions was analyzed for homology with human proteome and no epitope was found to be homologous with human proteome.

**Table 2.** Probable Antigenic Proteins ^a^

HCV Proteins	Antigenicity Threshold Level (0.4)	Antigenic Status
**Core**	0.3	Non-antigen
**gpE2**	0.49	Antigen
**NS3**	0.42	Antigen
**NS5A**	0.40	Antigen
**NS5B**	0.35	Non-antigen

^a^ Abbreviation: HCV, hepatitis C virus; gpE2, enveloped glycoprotein 2.

**Table 3.** B-Cell Epitopes and Their Conservation in Envelope Glycoprotein 2From Hepatitis C Virus 3a and Other Genotypes ^a,b^

Epitopes	Name (Antigenicity Score)	Position	Pakistan HCV 3a (gpE2)		Asia HCV 3a (gpE2)		World HCV 3a (gpE2)		World HCV (gpE2) 1-6 Genotypes	
			≥ 80%	≥ 90%	80%	≥ 90%	80%	≥ 90%	80%	≥ 90%
**QLVNTNGSWHIN**	B1 (0.81)	409-420	-	92	-	94	-	94	-	90
**TRG**	B2 (0.01)	647-649	-	100	-	100	-	100	-	100
**PCSFT**	B3 (0.9)	676-680	-	100	-	100	-	100	-	100
**MPAALSTGLIH **	B4 (0.75)	682-692	-	100	-	100	-	100	-	90
**RPPSGRW**	B5 (0.56)	544-550	-	94	-	90	-	92	-	-
**GAPPCNIYGGGGSLQN **	B6 (0.42)	566-581	-	92	-	90	-	92	-	-
**RCDIEDRDRSEQ**	B7 (1.72)	651-662	-	98	-	98	-	94	-	-
**STGCPQRLS**	B8 (0.21)	450-458	89	-	86	-	84	-	-	-
**WGPLTDANITGSSDDRPYCWHY**	B9 (1.2)	470-491	84	-	82	-	82	-	-	-
**APRPCGTVPASTVVCGPVYCFT **	B10 (0.3)	491-512	80	-	80	-	80	-	-	-
**FRKHPETTYSRCGAGPWL **	B11 (0.36)	586-603	82	-	80	-	80	-	-	-

^a^ Antigenicityscore less than 0.4 is considered non immunogenic.

^b^ Abbreviations: HCV, hepatitis C virus; gpE2, envelope glycoprotein 2.

**Table 4.** Class II MHC-Specific T-cell Epitopes Conservancy (%) in Envelope Glycoprotein 2 From Hepatitis C Virus 3a and Other Genotypes ^a,b^

Epitopes	Name (Antigenicity Score)	Alleles	Position	Pakistan HCV 3a (gpE2)		Asia HCV 3a (Egp2)		World HCV 3a (gpE2)		World HCV (gpE2) 1-6 Genotypes	
				≥80%	≥90	≥80%	≥90%	≥80%	≥90%	≥80%	≥90%
**WHINSTALN**	R1 (1.05)	44	420-428	-	100	-	98	-	98	-	90
**LSTGLIHLHQNIVD**	R2 (0.5)	44	685-698	-	100	-	100	-	100	-	100
**YCFTPSPVVVG**	R3 (1.7)	32	508-518	-	100	-	98	-	98	-	98
**FCPTDCFRK**	R6 (0.46)	16	580-588	-	100	-	100	-	100	-	98
**WHYAPRPCG**	S1 (0.23)	35	488-497	84	-	84	-	82	-	82	-
**VVVGTTDAR**	S2 (2)	19	515-523	84	-	88	-	89	-	80	-
**LKTCGAPPC**	S3 (-0.418)	36	562-570	89	-	87	-	87	-	80	-
**LQLVNTNGSWI**	S4 (0.72)	51	412-423	89	-	89	-	85	-	84	-

^a^ Antigenicityscoreless than 0.4 is considered non immunogenic.

^b^ Abbreviations: HCV, hepatitis C virus; gpE2, envelope glycoprotein 2.

**Table 5.** Class II MHC-Specific T-Cell Epitopes Conservancy (%) in Envelope Glycoprotein 2 From Hepatitis C Virus 3a and Other Genotypes ^a,b^

Epitopes	Name (Antigenicity Score)	Alleles	Position	Pakistan HCV 3a (gpE2)		Asia HCV 3a (gpE2)		World HCV 3a (gpE2)	
				≥80%	≥90%	≥80%	≥90%	≥80%	≥90%
**LPCSFTPMPALST**	Q1 (0.62)	35	675-687	-	100	-	100	-	100
**LAILPCSFT**	Q 2 (0.57)	34	672-680	-	100	-	100	-	100
**YLYGVGSGMVG **	Q 3 (0.58)	21	701-711	-	100	-	100	-	100
**WGPLTDANA **	Q 4 (0.4)	15	470-478	-	92	-	90	-	90
**LKWEFVILV**	Q 5 (1.66)	33	714-722	-	100	-	96	-	92
**VRMFVGGFEHRF **	Q 6 (0.6)	25	629-630	-	100	-	100	-	98
**WMNSTGFLK**	Q 7 (0.15)	19	555-563	-	98	-	90	-	90
**FNSTGCPQR**	Q 8 (0.5)	10	448-456	-	94	-	91	-	90
**LRPPSGRWF**	Q 9 (-2.3)	19	543-551	-	92	-	92	-	91
**WTRGERCDI**	Q 10 (0.9)	28	646-654	-	94	-	96	-	92
**LYGVGSGMVGWPLKWE**	Q 11 (1.25)	32	702-717	-	100	-	92	-	90
**ILPCSFTPM**	Q 12 (1.11)	10	674-682	-	98	-	98	-	98
**LLHSTTELA**	Q 13 (0.54)	26	765-773	-	100	-	100	-	100
**INTGFLAGL**	Q 14 (0.38)	8	434-442	-	90	-	90	-	90
**WLTPRCMVH**	Q 15 (0.8)	28	602-610	-	96	-	94	-	94
**YHRFNSTGC**	P1 (0.57)	26	445-453	80	-	80	-	81	-
**FKQGWGPLT**	P2 (1.36)	5	466-474	86	-	81	-	80	-
**LNCNESINT**	P3 (-0.17)	24	428-436	86	-	83	-	83	-
**FRKHPETTY**	P4 (0.29)	17	585-594	82	-	80	-	80	-
**MVGWALKWE**	P 5 (2.05)	26	709-716	89	-	86	-	84	-
**YYHRFNSTG**	P6 (0.98)	13	444-452	86	-	84	-	84	-

^a^ Antigenicityscoreless than 0.4 is considered non immunogenic.

^b^ Abbreviations: HCV, hepatitis C virus; gpE2, envelope glycoprotein 2.

**Table 6.** Class I MHC-Specific T-Cell Epitopes Conservancy (%) in Envelope Glycoprotein 2 Among Hepatitis C Virus3a and Other Genotypes ^a,b^

Epitopes	Names (Antigenicity score)	Alleles	Position	Pakistan HCV 3a (gpE2)	Asia HCV 3a (gpE2)	World HCV 3a (gpE2)	World HCV 1-6 Genotypes (gpE2)
**PLLHSTTEL**	M1 (0.40)	23	664-673	100	100	100	90
**SPVVVGTTD **	M2 (1.89)	9	512-520	100	100	100	100
**NESINTGFL**	M3 (0.98)	27	431-439	94	90	90	> 20

^a^ Antigenicityscore less than 0.4 is considered non immunogenic.

^b^ Abbreviations: HCV, hepatitis C virus; gpE2, envelope glycoprotein 2.

## 5. Discussion

High antigenicity of the HCV gpE2 is considered as the most impending obstacle to HCV vaccine (25). Finding the right antigenic determinants or epitopes that can induce important immune response against pathogen is the major challenge to developing HCV vaccine. New advancements in sequence databases and computer-based epitope design have been known to screen out all possible epitopes that are able to provoke immune response against a particular pathogen (26). This study was designed to predict the conserve B-cell-binding and class I as well as II MHC-binding epitopes in HCV gpE2 by using computer-based in silico approach. The HCV gpE2 is found to be more immunogenic in comparison to other protein when identified by VaxiJen using alignment independent algorithm as the in silico identification of antigens was above 0.4% of threshold level. Among B-cell epitopes, B1 to B4 epitopes were found to be conserved among 3a and other genotypes (1-6). The data showed that these epitopes might produce antibodies not only against HCV 3a genotypes but also against other genotypes. Recent in vitro studies have also confirmed that some of these epitopes produce neutralizing antibodies. Keck et al. reported that epitope present at position 410 to 425 (B1) produces neutralizing antibodies (15). Another study reported epitope I (B1) as highly conserved among the genotypes as well as the major antibody neutralization target (26). Human monoclonal antibody HCV1 also recognizes a highly-conserved linear epitope of the HCV gpE2 (amino acids 412-423) and neutralizes a broad range of HCV genotypes (27). Our In silico results are in line with this report as B1 was more than 90% conserved for all genotypes. These results are further supported by a recent report advocating B1, B3, and B4 as ideal B-cell epitopes with antigenicity ranging from 0.75 to 0.9 for 3a genotype (28); in addition, our study emphasized these epitopes as being universal for all major genotypes (Table 3). The epitopes B5 to B7 were ≥ 90% conserved whereas certain epitopes (B8 to B11) were found to be ≥ 80% conserved among HCV 3a population; these epitopes, however, were not conserved among other genotypes (Table 3). Furthermore, antigenicity of B10 and B11 was also low (< 0.4). On the other hand, B9 and B7 had very high antigenicity of 1.2 and 1.75, respectively, and as further supported by the recent report by Idrees et al. (28), these epitopes might be valuable for 3a genotype theraputics. Antibodies to amino acids 496 to 515 were isolated by affinity binding and elution from the serum of a vaccinated chimpanzee and were found to specifically neutralize chimeric 1a/2a, 1b/2a, and 2a HCV cell culture (29). In our study, B5 seems to be a part of the mentioned epitope and showed 80% conservation for other genotypes. These results provided evidence that broadly neutralizing antibodies to HCV might protect against heterologous viral infection and suggested that a prophylactic vaccine against HCV might be achievable.

Many neutralizing antibodies against HCV *gpE2* gene are reported; however these antibodies differ in their mechanism of neutralization and are mostly homologus in action (17). Recent studies have proved that predicted epitopes by different online tools shows immunogenicity when experimentally checked in herpes simplex virus, influenza A virus*,* and *Vibriomimicus *(30, 31). The human antibodies raised against HCV gpE2 epitopes do not offer protection against multiple viral infections; the preseason may be related to either genetic variations among viral strains particularly within the hyper variable region-1 (HVR-1), low titers of anti-gpE2 antibodies, or interference of non-neutralizing antibodies with the function of neutralizing antibodies (32). Thus, recombinant or synthetic antigens may be more efficient in inducing neutralizing antibodies to certain epitopes and screening virally infected patients may not be the best approach for finding new cross-reactive epitope.

Analysis of T-cell immune responses to gpE2 has been previously established. In silico-based immune dominant CD8+ epitopes selected for HLA-A2 and HLA-H2d had shown encouraging delayed-type hypersensitivity response in vaccinated mice (21). In this study, conserved class I and II MHC epitopes were predicted and their conservancy was checked in other genotypes. The initiative for this study was based on the information that certain T-cell epitopes on the HCV gpE2 play important role in viral clearance (33). Among T-cell epitopes, some epitopes showed ≥ 90% and ≥ 80% conservation among HCV 3a as well as other HCV genotypes. Many T-cell epitopes reported in this study showed maximum allele-binding affinity confirming them as a potential T-cell epitopes. M2 and M1 are found to be the best class I MHC epitope to be used for synthetic vaccine against multi-isotypes of HCV; R1, R3, and Q6 likewise are ideal class 2 MHC-specific epitopes with high antigenicity score and high conservancy across major genotypes. M2,Q5, and R3 are also predicted to be an ideal candidates for T-cell-based vaccine for HCV 3a genotype (29); however, we further suggested that M1, M2, and R3 were equally good for other genotypes as they are > 90% conserved across six major genotypes (Tables 4 and 5).

Thus, immune informatics tools were applied in the present study to predict the antigenicity of HCV 3a genotype gpE2 followed by prediction of its B-cell and T-cell epitope and conservancy of these epitopes among Pakistan, Asia, and other countries population infected with HCV 3a and other genotypes. In comparison to those epitopes derived from highly variable genome region, the use of conserved epitopes among protein could provide broader protection against HCV 3a and other genotypes. Therefore, these epitopes can be used as effective vaccine candidates for Asian and other continents residents.

This analysis showed that predicted epitopes can be used for vaccine design against HCV. Our results showed that most of B-cell and T-cell epitopes predicted from PKE2 showed higher conservation in 3a *gpE2* gene. The conservation was observed to be higher in Pakistan followed by Asia and other countries as compared to other genotypes. These epitopes are potential candidates for genotype-specific vaccine design. We also proposed few significantly cross-reactive epitopes that can be used for vaccine development and are expected to elicit strong immune response for 3a as well as other genotypes.
